# Early outcome of simplified total arch reconstruction under mild hypothermia (30–32 °C) with distal aortic perfusion

**DOI:** 10.1186/s13019-023-02448-2

**Published:** 2023-11-14

**Authors:** Hua-Jie Zheng, Xin Liu, Ping He, Jun Li, Xian-Pu Zhang, Yong-Bo Cheng, De-Qing Lin, Chao-Jun Yan, San-Jiu Yu, Wei Cheng

**Affiliations:** Department of Cardiac Surgery, Southwest Hospital, Third Military Medical University (Army Medical University, No. 30, Gaotanyan, Shapingba District, 400038 Chongqing, P.R. China

**Keywords:** Total arch reconstruction, Mild Hypothermia, Distal aortic perfusion, Moderate Hypothermia, Circulatory arrest

## Abstract

**Objective:**

We designed a simplified total arch reconstruction (s-TAR) technique which could be performed under mild hypothermia (30–32 °C) with distal aortic perfusion. This study aimed to compare its efficacy of organ protection with the conventional total arch reconstruction (c-TAR).

**Methods:**

We reviewed the clinical data of 195 patients who had ascending aortic aneurysm with extended aortic arch dilation and underwent simultaneous ascending aorta replacement and TAR procedure between January 2018 and December 2022 in our center. 105 received c-TAR under moderate hypothermia (25–28 °C) with circulatory arrest (c-TAR group); rest 90 received s-TAR under mild hypothermia (30–32 °C) with distal aortic perfusion (s-TAR group).

**Results:**

The s-TAR group demonstrated shorter CPB time, cross-clamp time and lower body circulatory arrest time compared with the c-TAR group. The 30-day mortality was 2.9% for the c-TAR group and 1.1% for the s-TAR group (*P* = 0.043). The mean duration of mechanical ventilation was shorter in the s-TAR group. Paraplegia was observed in 4 of 105 patients (3.8%) in the c-TAR group, while no such events were observed in the s-TAR group. The incidence of temporary neurologic dysfunction was significantly higher in the c-TAR group. The incidence of permanent neurologic dysfunction also showed a tendency to be higher in the c-TAR group, without statistical significance. Furthermore, the incidence of reoperation for bleeding were significantly lower in the s-TAR group. The rate of postoperative hepatic dysfunction and all grades of AKI was remarkably lower in the s-TAR group. The 3-year survival rate was 95.6% in the s-TAR group and 91.4% in the c-TAR group.

**Conclusions:**

s-TAR under mild hypothermia (30–32℃) with distal aortic perfusion is associated with lower mortality and morbidity, offering better neurological and visceral organ protection compared with c-TAR.

## Introduction

An ascending aortic aneurysm with extended aortic arch dilation is a serious condition that requires surgical intervention to prevent aortic rupture or dissection. The recommended treatment is simultaneous ascending aorta replacement and total arch reconstruction (TAR) [1]. TAR is initially developed with deep hypothermic (14.1–20.0℃) circulatory arrest (DHCA), which lasts 20–40 min on average [2]. Such a relatively long period of DHCA is associated with risks for different kinds of adverse complications that affect the overall survival of patients [3]. With the development of selective antegrade cerebral perfusion (SACP) for brain protection, it has allowed for TAR with moderate hypothermic (20.1–28.0℃) circulatory arrest (MHCA) [4, 5]. However, reported neurologic damage rates after the use of MHCA have been diverse: from 5.5–33.3% [6, 7]. As a result, some modified TAR techniques under mild hypothermia (28–34℃) have been developed for the purpose of organ protection [8–11].

We designed a simplified total arch reconstruction (s-TAR) technique which could be performed under mild hypothermia (30–32 °C) with distal aortic perfusion. This study aimed to compare its efficacy of organ protection with the c-TAR in the clinical practice.

## Methods

### Ethics statement

This study was approved by the Institutional Review Board of Southwest Hospital of Third Military Medical University (Army Medical University) KY2021058 and conducted in accordance with the Declaration of Helsinki (as revised in 2013). The Institutional Review Board of Southwest Hospital of Third Military Medical University (Army Medical University) waived the need for patient’s informed consent due to the retrospective nature of the study.

### Patient population

**Fig. 1:**
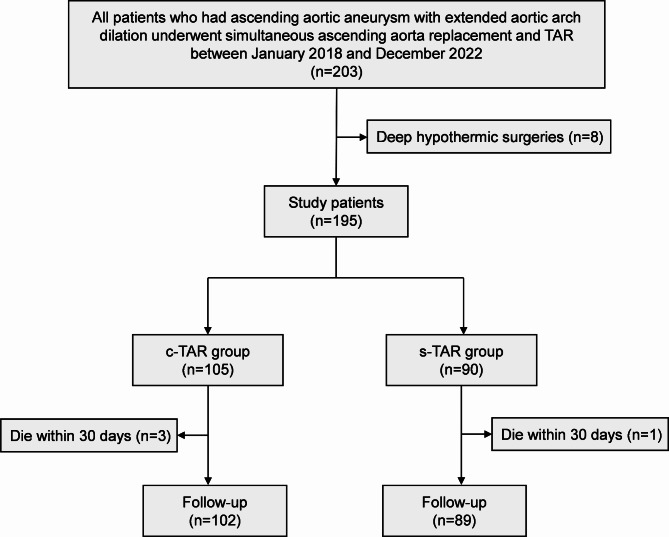
Study flow chart. c-TAR, conventional total arch reconstruction; s-TAR, simplified total arch reconstruction

Between January 2018 and December 2022, 203 consecutive patients who had ascending aortic aneurysm with extended aortic arch dilation underwent simultaneous ascending aorta replacement and TAR procedure in our center, of which 8 patients who had received deep hypothermic surgeries were excluded (Fig. 1). Among the remaining 195 patients, 105 received c-TAR under moderate hypothermia (25–28 °C) with circulatory arrest (c-TAR group); rest 90 received s-TAR under mild hypothermia (30–32 °C) with distal aortic perfusion (s-TAR group). The indication for intervention was maximum diameter of ascending aorta > 55 mm and aortic arch in zone II > 35 mm.

All procedures were performed by two dedicated surgeons. The decision to proceed with s-TAR or c-TAR was discretionary based on the underlying clinical condition. In general, for each one patient from the s-TAR cohort, one control subject was recruited into the c-TAR cohort. The 1-to-1 matching was based on variables identified a priori to be of interest. Matching variables included age (± 5 years), sex (exact), weight (± 20 kg), height (± 20 cm), and left ventricular ejection fraction (LVEF, ± 10%).

### Indications of the s-TAR technique

The s-TAR technique was indicated for all patients admitted for TAR treatment after screening for the following exclusion factors: (i) primary tear involving the aortic arch or orifices of the three supra-aortic branches; (ii) severe atherosclerosis in the aortic arch or in the origin of supra-aortic vessels; and (iii) serious comorbidities such as ruptured aneurysm and severe coagulation dysfunction.

**Stent Grafts.** The stent graft (Microport Medical Co, Ltd, Shanghai, China), with different diameters (28–30 mm), comprises a 10-mm stent-free vascular graft on both ends, to which a conventional hand-sewn anastomosis can be performed. We used the one-branch vascular prosthesis (Maquet Cardiovascular, Wayne, NJ, USA) for ascending aorta replacement (diameter, 26–30 mm), and a 4-branched arch graft (Maquet Cardiovascular, Wayne, NJ, USA) for the c-TAR.

### Surgical procedures

Under general anesthesia, right radial and left femoral artery pressure, central vein pressure, nasal and rectal temperatures, and cerebral oximeter were monitored. After median sternotomy, cardiopulmonary bypass (CPB) was established by cannulating the femoral artery and placing a dual-stage atriocaval cannula in the right atrium. During the cooling phase, the ascending aorta (aortic root or valve in some patients) was replaced and other concomitant procedures were done if indicated. Upon reaching the target temperature, 500 mg of thiopental was administered, the patient’s head was packed in ice.

### s-TAR technique

**Fig. 2:**
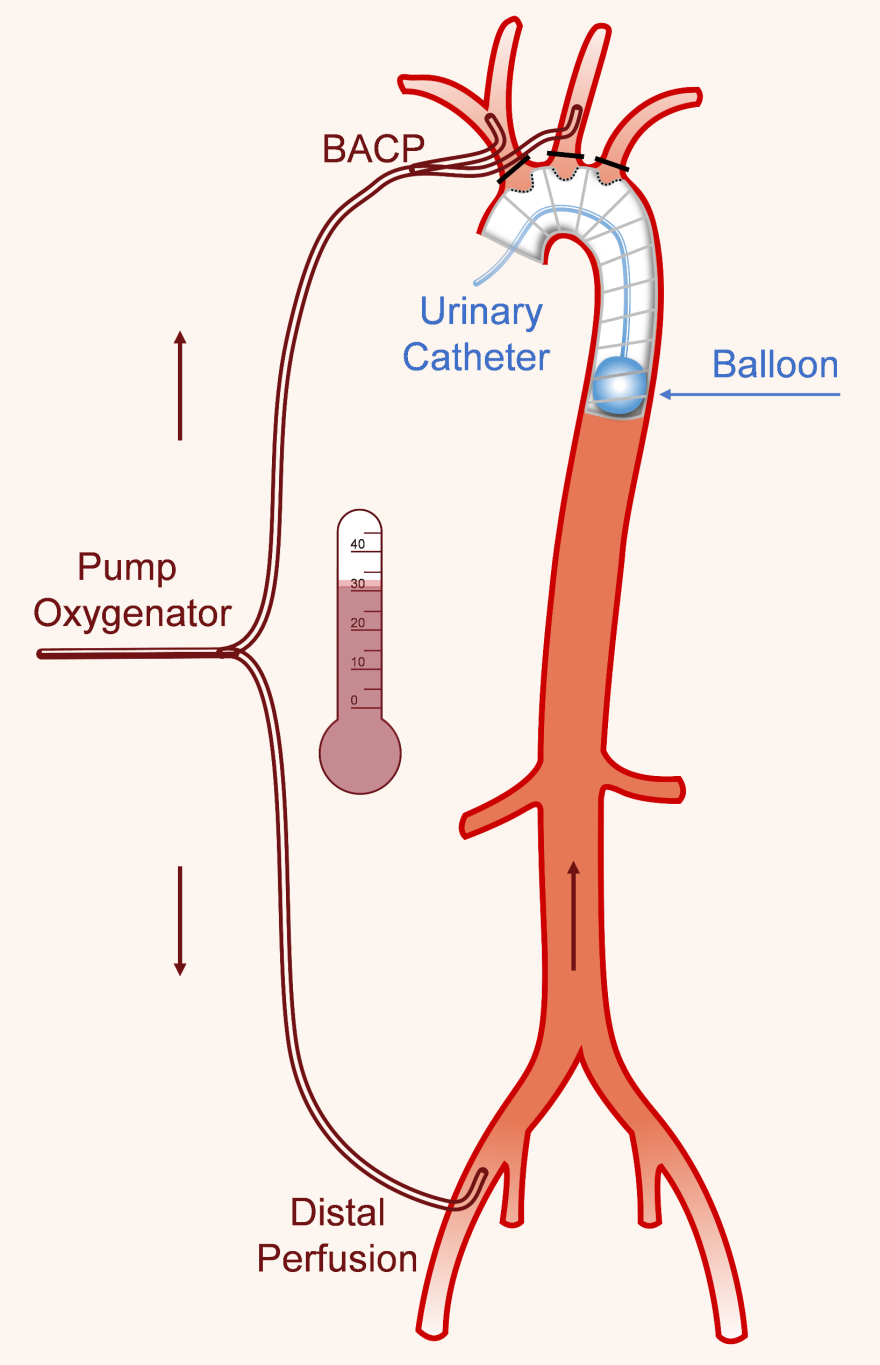
Schematic diagram of the s-TAR technique. s-TAR, simplified total arch reconstruction

Figure 2 showed a schematic representation of the s-TAR technique.

**Fig. 3:**
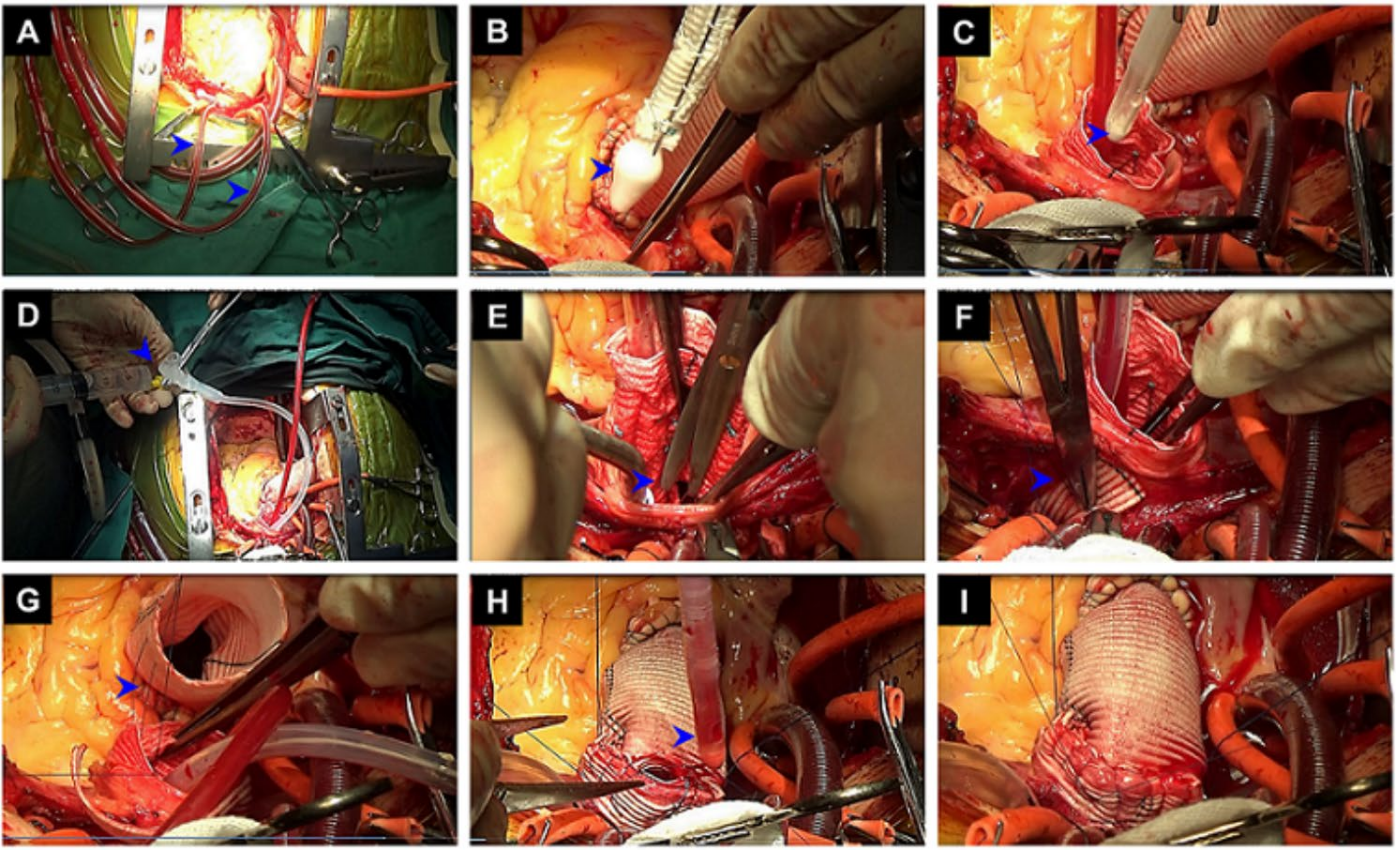
Each step of the s-TAR technique. **(A)** When the nasal temperature reached 30–32 °C, BACP (blue arrow) was initiated. **(B)** The stent graft (blue arrow) was deployed in the descending aorta. **(C) **A two-cavity urinary catheter (blue arrow) was deployed into the descending aorta. **(D)** The balloon was filled with about 30 ml saline by a syringe. **(E)** Three elliptical patches (blue arrow) on the polyester fabric of the stent graft were separately removed around each arch branch orifice. **(F)** The stent-free sewing fabric edge at the base of the modification was circumferentially sutured to the base of the respective branch vessels (blue arrow). **(G)** The end-to-end anastomosis (blue arrow) between the proximal aortic arch and the ascending aortic prosthesis was performed. (**H** and **I**) At the end of the suture, the femoral arterial line was temporarily clamped, and the aortic balloon was deflated and removed (blue arrow). s-TAR, simplified total arch reconstruction

**Fig. 4:**
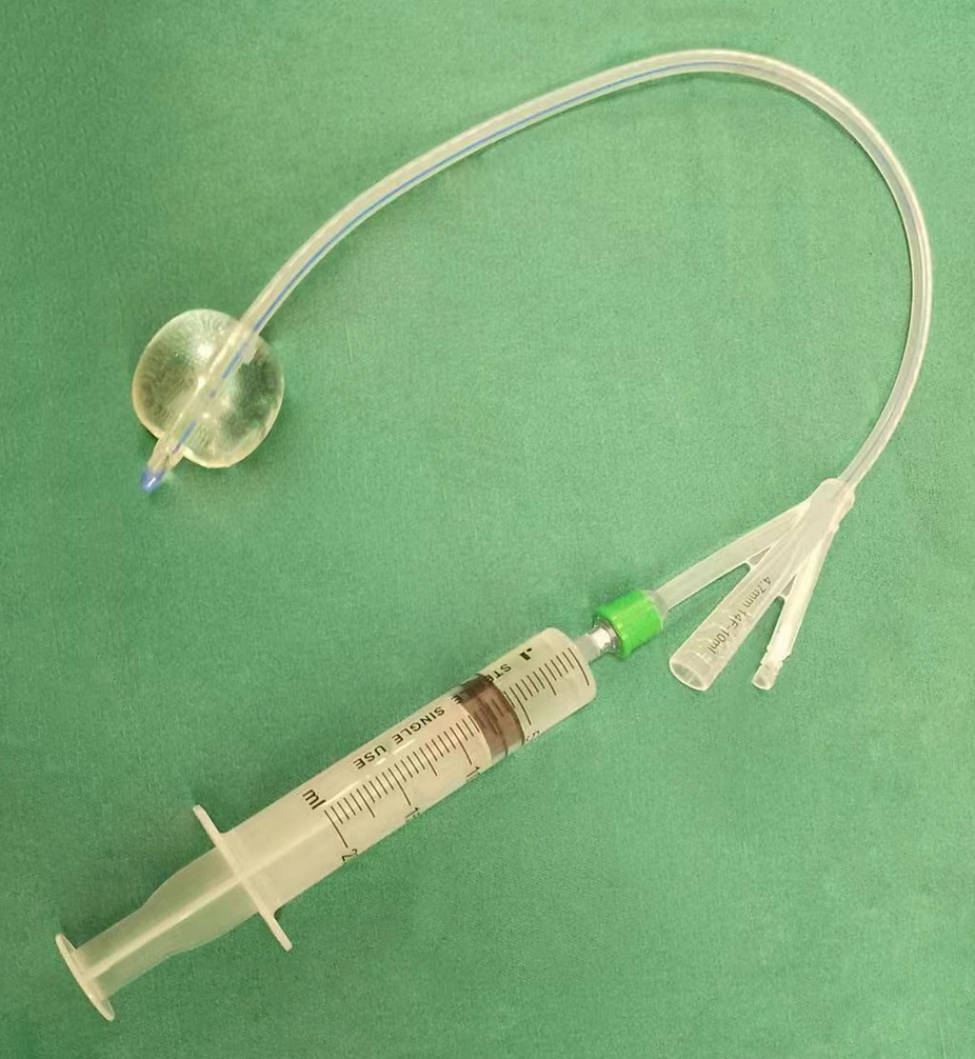
A two-cavity adult urinary catheter with a balloon at the tip

**Fig. 5:**
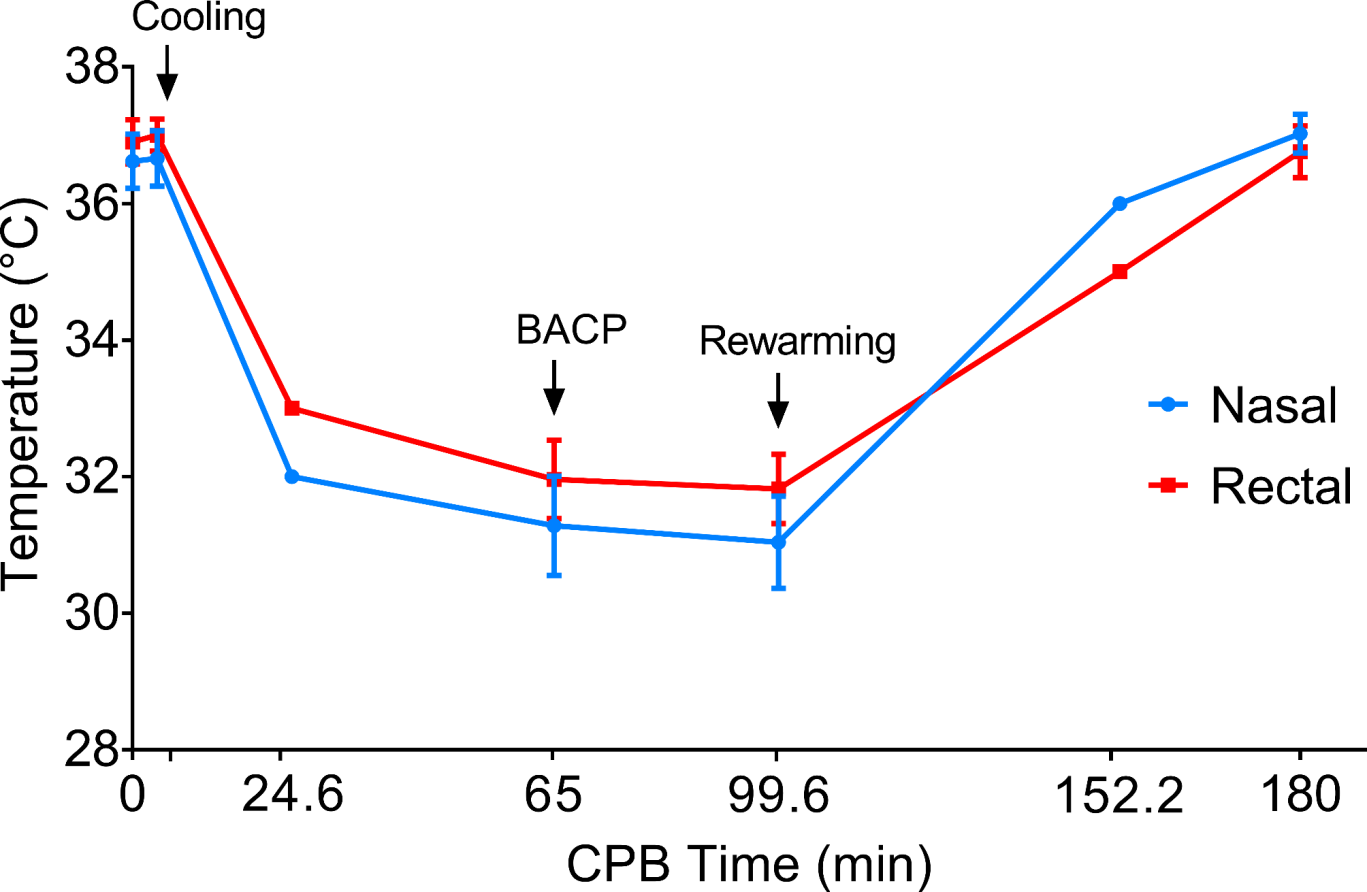
Change of temperature during the CPB of s-TAR procedure. Cooling was started after CPB reached total flow. During the cooling phase, the ascending aorta (aortic root or valve in some patients) was replaced and other concomitant procedures were done. Then, BACP was started when nasal and rectal temperatures reached 31.2 ± 1.0℃ and 32.3 ± 1.0℃, respectively. After the s-TAR procedure, rewarming started at 99.6 ± 25.17 min of CPB and took 52.6 ± 17.3 min for the rectal temperature to warm up to 35℃. After rewarming, patients were gradually weaned off CPB. CPB: cardiopulmonary bypass; BACP: bilateral antegrade cerebral perfusion; s-TAR, simplified total arch reconstruction

1. When the nasal temperature reached 30–32 °C, bilateral antegrade cerebral perfusion (BACP) was initiated. The setting of BACP considered an 8–10 ml/kg/min flow through the innominate artery and left common carotid artery (Fig. 3A). BACP has been our default approach because it is quick and easy and does not add time to the operation.

2. The stent graft was deployed in the descending aorta, and the proximal metal end of the stent graft was positioned just proximal to the origin of the innominate artery (Fig. 3B).
3. A two-cavity adult urinary catheter (20–22 Fr, Lusheng Medical Co, Ltd, Shandong, China) (Fig. 4) was deployed into the descending aorta (Fig. 3C). This was only for the s-TAR group.

4. The balloon was fully inflated with about 30 ml saline by a syringe, and positioned at the metal part of the stent graft (Fig. 3D). Perfusion of the lower body was resumed through the femoral artery.
5. Three elliptical holes on the polyester fabric of the stent graft were separately modified around each arch branch orifice under direct visualization and using a pair of surgical scissors. The modification diameter at the stent graft was similar to that of each branch orifice (Fig. 3E).
6. The polyester fabric of the stent graft at the base of the modification was sutured to the native aortic arch wall around each arch branch orifice using a continuous suture. Usually, the left subclavian artery (LSA) was reconstructed first, followed by the left common carotid artery and then the innominate artery (Fig. 3F).
7. The end-to-end anastomosis between the proximal aortic arch containing the intraluminal stent graft and the ascending aortic prosthesis was performed (Fig. 3G).
8. At the end of the suture, the femoral arterial line was temporarily clamped and the occlusive balloon was deflated and removed (Fig. 3H and I).
9. Cerebral perfusion was discontinued, and systemic circulation was gradually restarted. After rewarming, patients were gradually weaned off CPB (Fig. 5). The remainder of the procedures, including hemostasis and sternal closure, were performed routinely.

### c-TAR technique

The c-TAR technique was based on classical Sun’s procedure [12]. The procedure differed from the s-TAR technique in the following ways.

1. Our default setting was unilateral antegrade cerebral perfusion (UACP) through the right axillary artery when the nasal temperature reached 25–28 °C. Even if UACP was primarily planned, the conversion of UACP to BACP was initiated by the insertion of a separate cannula into the left common carotid artery when cerebral oximeter readings fell more than 15% of baseline.
2. The anastomosis of the 4-branched graft and descending aorta was performed with circulatory arrest of the lower body.
3. When the distal anastomosis was completed, perfusion of the lower body was resumed through the perfusion limb of the 4-branched arch graft.

### Follow-up

Each patient underwent clinical examination, laboratory testing, and transthoracic echocardiography and computed tomography angiography (CTA) before discharge (~ 2 week postoperatively), 3 months after discharge, and every 6 months thereafter.

## End points

The primary end points were any-cause death and neurologic morbidity. Temporary neurologic dysfunction (TND) was defined as the presence of reversible postoperative motor deficit, confusion, agitation, or transient delirium. Computed tomography findings were required to be normal, with resolution of all symptoms before discharge. Permanent neurologic dysfunction (PND) was defined as the presence of new focal (stroke) or global (coma) permanent neurologic dysfunction caused by cerebral infarction or hemorrhage.

Second endpoints included reoperation for bleeding, paraplegia, hepatic dysfunction and acute kidney injury (AKI). Paraplegia was defined as muscle strength of lower limb ≤ grade 3 (able to resist gravity but not resistance). Hepatic dysfunction was defined as the peak value of aspartate aminotransferase or alanine aminotransferase exceeding 100 IU/L within 48 h after surgery. AKI was diagnosed and categorized as serum creatinine level increased to > 1.5 times the upper range of normal (133 µmol/l, > 1.5 times as > 200 µmol/l) (grade I), 2–3 times (grade II), > 3 times (grade III) and hemodialysis (grade IV) in the first 7 days after surgery. AKI was staged for severity according to a modified RIFLE criterion.

### Statistical analysis

Continuous variables are shown as mean ± standard deviation (SD) or as median (interquartile range: 25th to 75th percentile) in cases of skewed distribution. Categorical variables are presented as raw counts and percentages. Assessment of normality was performed using the Shapiro-Wilk test. For normally distributed continuous data, Student’s *t* tests were used. For non-normally distributed data, Mann-Whitney *U*-test was used. Categorical variables are presented as percentages. Pearson’s *χ*2 or Fisher’s exact test was used for categorical variables. Survival was estimated with the Kaplan-Meier method, and differences were compared with a log-rank test. A *P* value < 0.05 was considered significant. Analyses were performed using the statistical packages SAS version 9.4 (SAS Institute, Cary, North Carolina).

## Results

### Baseline characteristics

The c-TAR and s-TAR groups were well matched in baseline characteristics. No inter-group differences were found for demographics and comorbidities (Table 1).

**Table 1:** Preoperative characteristics

Variables	Total (n = 195)	c-TAR (n = 105)	s-TAR (n = 90)	*P* value
Demographics				
Male gender	137 (70.3)	75 (71.4)	62 (68.9)	0.857
Age (years)	51.9 ± 11.6	49.5 ± 12.8	53.7 ± 9.9	0.663
Body mass index (kg/m^2^)	26.7 ± 3.8	26.4 ± 3.7	26.9 ± 4.4	0.649
Comorbidities				
Hypertension	128 (65.6)	66 (62.9)	62 (68.9)	0.815
Diabetes mellitus	13 (6.7)	6 (5.7)	7 (7.8)	0.707
Marfan syndrome	13 (6.7)	7 (6.7)	6 (6.7)	0.762
COPD	10 (5.1)	5 (4.8)	5 (5.6)	0.652
Coronary artery disease	14 (7.2)	7 (6.7)	7 (7.8)	0.404
Renal dysfunction	5 (2.6)	3 (2.9)	2 (2.2)	0.451
Liver dysfunction	4 (2.1)	2 (1.9)	2 (2.2)	0.522
Congestive heart failure (NYHA ≥ III)	5 (2.6)	2 (1.9)	3 (3.3)	0.669
History of stroke/TIA	6 (3.1)	3 (2.9)	3 (3.3)	0.749
Previous cardiac surgery	3 (1.5)	1 (1.0)	2 (2.2)	0.835

Continuous data are presented as the mean ± SD, and categorical data as number (%).

COPD, chronic obstructive pulmonary disease; NYHA, New York Heart Association; TIA, transient ischemic attack; c-TAR, conventional total arch reconstruction; s-TAR, simplified total arch reconstruction; SD, standard deviation.

### Operative details

The operative details are presented in Table 2. No patient died intraoperatively. No significant differences in the rate of concomitant procedures were found in both groups. The s-TAR group demonstrated shorter CPB time, cross-clamp time, and lower body circulatory arrest time. Accordingly, BACP started when the intraoperative nasal and rectal temperature reached 31.2 ± 1.0℃ and 32.3 ± 1.0℃ in the s-TAR group, respectively.

**Table 2:** Operative details

Variables	Total (n = 195)	c-TAR (n = 105)	s-TAR (n = 90)	*P* value
Concomitant procedures				
Bentall procedure	119 (61.0)	62 (59.0)	57 (63.3)	0.656
Wheat procedure	57 (29.2)	32 (30.5)	25 (27.8)	0.795
David procedure	19 (9.7)	11 (10.5)	8 (8.9)	0.718
CABG	6 (3.1)	3 (2.9)	3 (3.3)	0.602
Mitral valve replacement	9 (4.6)	4 (3.8)	5 (5.6)	0.436
Operation time				
CPB time (min)	195.6 ± 54.7	210.2 ± 59.8	176.5 ± 52.3	0.038
Cross-clamp time (min)	126.7 ± 37.5	135.6 ± 38.2	104.6 ± 33.8	0.042
Lower body circulatory arrest time (min)	16.9 ± 3.7	22.4 ± 3.9	7.8 ± 2.2	0.015
Lowest nasal temperature (°C)	28.1 ± 1.6	26.2 ± 1.0	31.2 ± 1.0	0.031
Lowest rectal temperature (°C)	29.4 ± 1.7	27.4 ± 1.0	32.3 ± 1.0	0.034

Continuous data are presented as the mean ± SD, and categorical data as number (%).

CABG, coronary artery bypass grafting; CPB, cardiopulmonary bypass; c-TAR, conventional total arch reconstruction; s-TAR, simplified total arch reconstruction; SD, standard deviation.

### Perioperative outcomes

The perioperative outcomes are summarized in Table 3. The 30-day mortality was 2.9% for the c-TAR group and 1.1% for the s-TAR group (*P* = 0.043). In the c-TAR group, causes of death were diffuse coagulopathy in 2 patients and multiple organ failure in 1 patient. In the s-TAR group, causes of death was low cardiac output syndrome in 1 patient. The s-TAR group had shorter intensive care unit (ICU) stay, shorter postoperative hospital stay, and lower total hospitalization costs than the c-TAR group.

**Table 3:** Perioperative Outcomes

Variables	Total (n = 195)	c-TAR (n = 105)	s-TAR (n = 90)	*P* value
30-day mortality	4 (2.1)	3 (2.9)	1 (1.1)	0.043
ICU length of stay (hours)	103.8 ± 23.9	125.7 ± 22.3	86.6 ± 24.4	0.037
Postoperative hospital stay (days)	11.6 ± 2.3	14.6 ± 2.7	8.9 ± 2.6	0.034
Total hospitalization cost (×10^5^, yuan)	16.8 ± 3.5	19.3 ± 3.5	14.2 ± 3.6	0.041
TND	12 (6.2)	9 (8.6)	3 (3.3)	0.018
PND	3 (1.5)	2 (1.9)	1 (1.1)	0.523
Ventilation time (hours)	45.4 ± 13.3	55.7 ± 13.8	32.9 ± 12.6	0.028
Reoperation for bleeding	4 (2.1)	3 (2.9)	1 (1.1)	0.026
Paraplegia	4 (2.1)	4 (3.8)	0	0.034
Hepatic dysfunction	26 (13.3)	18 (17.1)	8 (8.9)	0.028
AKI				
All AKI cases	50 (25.6)	35 (33.3)	15 (16.7)	0.029
Grade I	19 (9.7)	14 (13.3)	5 (5.6)	0.028
Grade II	20 (10.3)	13 (12.4)	7 (7.8)	0.034
Dialysis (Grade III&IV)	11 (5.6)	8 (7.6)	3 (3.3)	0.031
Perioperative transfusion (48 h)				
Packed red blood cells (units)	4.3 ± 2.3	5.7 ± 3.3	3.6 ± 2.2	0.042
Fresh-frozen plasma (units)	4.2 ± 2.6	5.2 ± 2.7	3.3 ± 2.5	0.047
Apheresis platelets (doses)	1.5 ± 0.7	2.5 ± 0.9	1.2 ± 0.8	0.029
Pooled cryoprecipitate (doses)	2.1 ± 1.0	2.9 ± 1.3	1.4 ± 0.8	0.031

Continuous data are presented as the mean ± SD, and categorical data as number (%).

ICU, intensive care unit; TND, temporary neurologic dysfunction; PND, permanent neurologic dysfunction; c-TAR, conventional total arch reconstruction; s-TAR, simplified total arch reconstruction; SD, standard deviation.

**Fig. 6:**
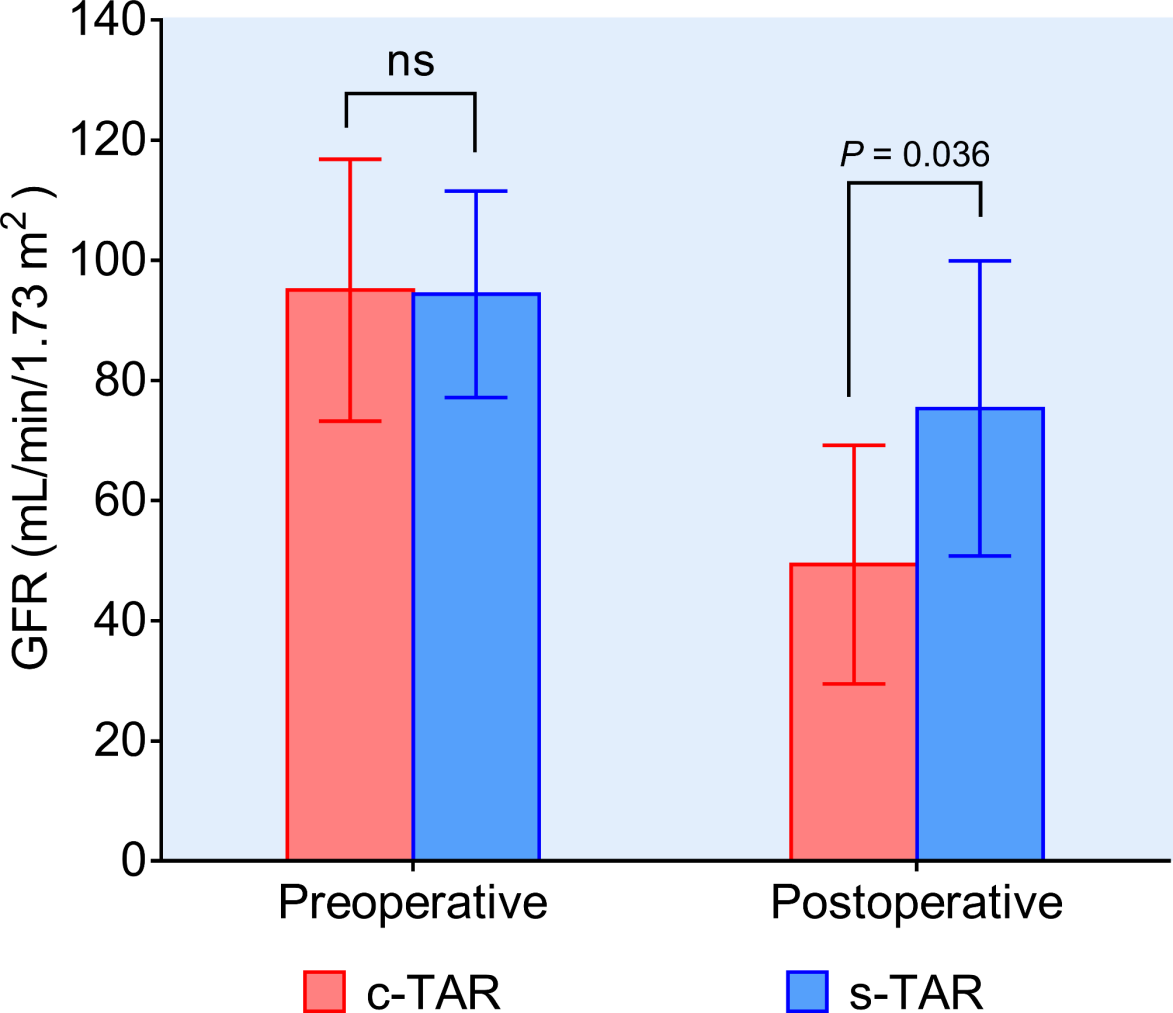
Preoperative GFR and lowest GFR in the first 7 days after surgery in the c-TAR and s-TAR groups. GFR, glomerular filtration rate; c-TAR, conventional total arch reconstruction; s-TAR, simplified total arch reconstruction

The mean duration of postoperative mechanical ventilation was shorter in the s-TAR group (32.9 ± 12.6 h vs. 55.7 ± 13.8 h; *P* = 0.028). Paraplegia was observed in 4 of 105 patients (3.8%) in the c-TAR group, while no such events were observed in the s-TAR group. The incidence of TND was also significantly higher in the c-TAR group. The incidence of PND showed a tendency to be higher in the c-TAR group, but there was no statistical significance. Furthermore, the incidence of reoperation for bleeding were significantly higher in the c-TAR group, which all resulted from surgical field errhysis due to coagulation dysfunction. Accordingly, patients in the c-TAR group accepted more packed red blood cells (5.7 ± 3.3 vs. 3.6 ± 2.2; *P* = 0.042), fresh-frozen plasma (5.2 ± 2.7 vs. 3.3 ± 2.5; *P* = 0.047), apheresis platelets (2.5 ± 0.9 vs. 1.2 ± 0.8; *P* = 0.029), and cryoprecipitate (2.9 ± 1.3 vs. 1.4 ± 0.8; *P* = 0.031) transfusion than patients in the s-TAR group. The rate of postoperative hepatic dysfunction and all grades of AKI was significant lower in the s-TAR group. In addition, postoperative lowest GFR in the first 7 days after surgery was higher in the s-TAR group (75.8 ± 23.5 vs. 49.5 ± 25.8; *P* = 0.036) (Fig. 6). Before discharge, all patients underwent CTA that confirmed correct positioning of the vascular graft without stenosis, endoleak or dissection around the anastomosis sites in both groups.

### Overall survival

**Fig. 7:**
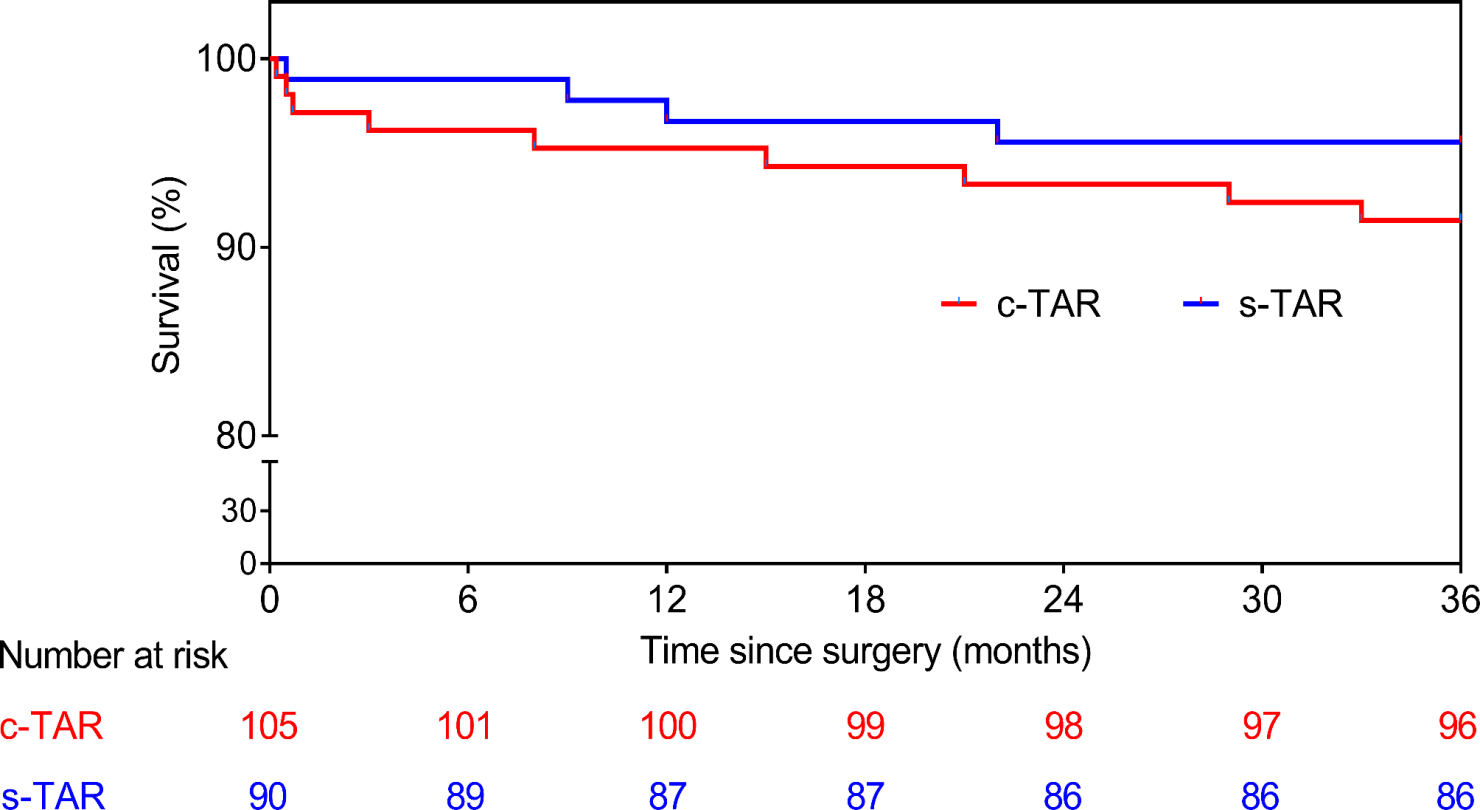
Kaplan-Meier survival curves for the c-TAR and s-TAR groups. c-TAR, conventional total arch reconstruction; s-TAR, simplified total arch reconstruction

All patients were followed postoperatively up to April 2023 by telephone or direct interview (no lost at follow-up). The average follow-up length was similar in both groups (3.2 ± 1.5 years for the c-TAR group and 3.3 ± 1.6 years for the s-TAR group). Kaplan-Meier survival curves in Fig. 7 showed the 3-year survival rate was 95.6% in the s-TAR group and 91.4% in the c-TAR group. There was no aortic-related death, and no patient had a new neurologic dysfunction, paraplegia, or aortic-related reintervention at the time of analysis.

## Discussion

With our s-TAR technique, the lowest nasal temperature was raised to about 31℃ and the lower body circulatory arrest time was shortened to about 7.8 min. Compared with the c-TAR group, the incidence of adverse events was remarkably lower in the s-TAR group, such as 30-day mortality, post-operative neurologic dysfunction, prolonged ventilation, reoperation for bleeding, paraplegia, hepatic dysfunction and AKI. Overall survival was significantly improved in the s-TAR group.

Among healthy adults in China, the normal diameter of aortic arch is about 23.9–29.8 mm. A 50% increase is referred to about 35–45 mm, which is considered arch aneurysmal dilatation. In the presence of ascending aortic aneurysm, the diameter of aortic arch in zone II > 35 mm could be considered an indication for concomitant aortic arch reconstruction. If the ascending aorta is replaced alone, the residual intact arch may be at excess risk of aortic dilation, dissection, and thus reoperation. Therefore, prophylactic reconstruction of the intact aortic arch is necessary as long as the patient’s condition permits this. Given the remnant aortic pathologies after hemi-arch replacement, this procedure may potentially place the patients at excess risk of aortic dilation, dissection, rupture and thus reoperation. Therefore, our center has not used this method since 2015. The Sun’s procedure involves the separate anastomoses of the three supra-aortic branches and 4-branched arch graft and implantation of a stent graft into descending aorta, which has still been used in our center.

If the diameter discrepancy between stent graft and zone II of the aortic arch was large, the s-TAR procedure could still be performed. When there is constant blood flow through the aortic arch, it creates pressure on the aortic wall and dilates the aortic arch. However, when the proximal end of the aortic aorta is blocked during the operation, the dilated aortic arch would shrink back. Thus, the implanted stent graft could be tightly attached to the wall of the native aortic arch. Follow-up data in this study suggested that the stent graft was attached well with the native aortic arch.

The s-TAR technique provides several advantages over c-TAR technique. First, the s-TAR technique preserves the integrity of the aortic arch, and the entire procedure is performed within the aortic arch itself; second, the stent graft implantation, modification and suture is easily completed in 6 to 8 min; third, it does not require replacement of the three brachiocephalic vessels, reducing the number of anastomosis sites; four, it does not require distal end to-end anastomosis, reducing bleeding risk; and finally, the lower body circulatory arrest time was dramatically decreased, reducing the risk of ischemic injury of spinal cord and visceral organ.

The balloons size (about 30 ml) of an adult urinary catheter were well matched the size of the stent graft of the aorta (26–30 mm). The inner face of the stent graft is smooth enough so the inflated balloon does not get punctured. Consequently, the distal end could achieve complete closure when the balloon is adequately inflated and positioned at the metal part of stent graft. In some cases, there is a little back bleeding around the balloon. This problem could be resolved by replenishing the balloon with the proper amount of saline to maintain its filled state. In other cases, blood backflow may occur from the abdominal aorta. This could be solved by removing the blood with strong extracardiac suction or adjusting the flow rate from CPB temporally in the case of a large amount of back bleeding.

During aortic arch surgeries, high morbidity and mortality rates have persisted and may be secondary to profound hypothermia [13]. Consequently, we have seen a paradigm shift towards warmer temperatures with positive neurological outcomes being observed [14, 15]. Zierer and colleagues [16] applied warmer circulatory arrest with mild hypothermia (average, 30.5 °C) and reported surgical outcomes that showed a 6% rate of PND, an 8% rate of 30-day mortality, and 1 patient with paraplegia. The authors concluded selective cerebral perfusion with mild hypothermia offered sufficient cerebral and distal organ protection. With warmer temperature, the most concerning thing is visceral and spinal cord protection. Experimental animal studies have showed that the safety limit for the spinal cord was 90 min at 28 °C and 60 min at 32 °C [17, 18]. According to these reports, cerebral and visceral protections during aortic arch repair could be safely performed with mild hypothermia and selective cerebral perfusion. From our results, visceral organ function and overall survival was maintained significantly better in the s-TAR group. Therefore, we may conclude that temperature could be increased to the mild hypothermic level (30–32 °C) without compromising visceral organ protection.

The method of cerebral perfusion during the s-TAR technique was BACP, while UACP was mainly used in the c-TAR technique, converting to BACP only when deemed necessary. Many studies comparing UACP to BACP demonstrated that UACP offered as much cerebral and visceral organ protection as BACP [19, 20]. Due to the circle of Willis, UACP through the right axillary artery can also perfuse into the vertebral artery, the internal carotid artery, and the basilar artery, allowing entire cerebral perfusion [21]. However, 6–17% of patients present with an incomplete circle of Willis [22]. Therefore, BACP can theoretically provide more sufficient cerebral perfusion than UACP.

In our study, the transfusion requirements increased significantly and the rate of reoperation for bleeding was significantly higher in the c-TAR group. Previous studies reported that moderate hypothermia may reduce the activity of enzymes involved in platelet activation pathways and clotting factors, both of which could increase transfusion requirements and bleeding complications [23]. In addition, prolonged CPB time also independently predicted reoperation for bleeding because prolonged CPB could signify a more complex and prolonged operation [24]. However, the s-TAR technique successfully raised the lowest hypothermic cardiopulmonary bypass temperature and shortened the time of CPB, which effectively reduced the risk of the coagulation disturbance and reoperation for bleeding.

A growing body of evidence suggests that perioperative transfusions of large amounts of red blood cells are associated with an increased incidence of AKI after cardiac surgery [25]. The association between transfused red blood cells and AKI may be related to impaired oxygen delivery, decreased deformability of stored red blood cells, prothrombotic effects from the increased release of procoagulant factors, and transfusion-related immunosuppression. In particular, transfusion of several units of older stored red blood cells can lead to high levels of circulating free haemoglobin and iron [26]. It is estimated that the transfusion of two units of red blood cells can increase plasma free haemoglobin to 10 times the normal level [27]. Free haemoglobin and iron have obviously toxic effects on the kidneys.

### Study limitations

This study has some limitations. First, this is a retrospective, and single-institutional study. Second, the surgeon’s preference could be a potential bias of this study. Further research with multiple centers and larger samples is scheduled.

## Conclusions

s-TAR under mild hypothermia (30–32℃) with distal aortic perfusion is associated with lower mortality and morbidity, offering better neurological and visceral organ protection compared with c-TAR.

## Data Availability

The data that support the findings of this study are available from the corresponding authors upon reasonable request.
